# Notes and News

**Published:** 1887-07-23

**Authors:** 


### NOTES AND NEWS.

COLLECTED AND COMMUNICATED.

Dr. Hodgson and Mr. Maitland have been appointed assistant-surgeons to the Oldham Infirmary.

At the Throne Hospital, Belfast, Miss Bostock, an English lady, has been appointed to the position of Lady Superintendent in succession to Miss Shields. Miss Bostock will enter upon the duties of the office in the beginning of September.

The Cleveland Convalescent Home is in want of £250 for the payment of its rent. It is a pity that at the very period when the Home is most used by the people it should be burdened with a debt. Will no good Samaritan come forward with the required amount?

On Saturday evening, at the Blackburn Infirmary, a concert was given by the members of the choir of St. Joseph's Church, Darwen. The various songs and pieces were well rendered, and gave much pleasure to the inmates.

The annual meeting of "The After-Care Association for Poor and Friendless Female Convalescents upon Leaving Asylums for the Insane," was held on Thursday last, at 83, Lancaster Gate, W. In the absence of the President (Earl of Meath), Dr. Hack Tuke took the chair. Amongst those who sent letters of apology for non-attendance were the Archbishop of Canterbury, Cardinal Manning, Archdeacon of Middlesex, Dr. Lockhart Robertson, Dr. E. Sheppard, Dr. G. Stanley Elliot, T. Salt, Esq., M.P. The meeting was addressed by Rev. C. J. Ridgway, Rev. E. S. Dewick, Dr. G. H. Savage, Dr. J. Rees Philipps, Dr. T. C. Shaw, and others. A fair amount of progress has been made during the past year, but funds are much required to extend the work of this valuable Society. Full particulars may be obtained of the Secretary, Mr. H. Thornhill Roxby, Emblewood, Osbaldeston Road, London, N.

Major Ross, M.P., and Major-General Keatinge, C.S.I., V.C., have been reappointed Chairman and Deputy Chairman respectively of the Council of The Hospitals Association for the ensuing year, to which Messrs. E. F. Coates, of Epsom and Ewell Cottage Hospital, and Mr. A. H. Haggard, Secretary of the London Hospital, have been added, the latter in lieu of Mr. W. J. Nixon, resigned.

The Executive Committee has been reappointed for the ensuing year, with the substitution of Sir Edmund Hay Currie, Dr. Steele (Guy's), and Dr. Rayner (Hanwell Asylum), for Mr. Almond Hind, Mr. W. J. Nixon, and Dr. Theodore Williams.

The Sectional Committee on Nursing and Domestic Management (consisting of ladies having charge as lady superintendents or matrons of most of the leading institutions) has been reappointed.

The Council of The Hospitals Association has decided that the evening meetings of the Association shall be held on the second Wednesday of each month at eight p.m., and as already several papers on subjects of great importance in connection with Hospitals and Medical Charities have been promised, it is hoped that members will note the dates, and will attend in large numbers at the meetings. The meeting held last session at the Brompton Consumption Hospital having proved so successful, it is intended to hold several of, if not all, the meetings of next session at other hospitals of the metropolis, if arrangements can (as it is anticipated) be made for the consent of the managing bodies.

Members of The Hospitals Association are reminded that The Hospital, the Journal of the Association, will contain each month during the session copies of all papers read at the evening meetings, together with a report of the discussions thereon, so that those who live in the country, or who are otherwise prevented from being present, are informed of what takes place at the meetings at which their fellow-members are present.

Some time ago a gentleman from New York gave a sum of money as a "thank-offering" to the Torbay Hospital, to be used for the benefit of the patients. A sufficient amount was retained (the rest having been mostly spent in drives) to give a pic-nic on Paignton sands last Thursday week. At 1.30 a party of forty started in high spirits, and on arriving at the pier were joined by a few old friends, who had shortly before left the hospital wards. The day was lovely, the tea good and abundant, and the company very happy. The pier manager and his wife were most kind and attentive, and they, together with Mr. John Brown, spared no pains in helping the nurses to serve the tea. Mr. Webb supplied the carriages, with the exception of one midge. The gentleman who gave so much pleasure is entirely unknown to the hospital authorities, therefore the greater kindness on his part. They trust another year some other "stranger" may follow his example. More is left undone by want of thought than want of heart, and an act of kindness such as this is worth much more than all the talk we so often hear.

THE following vacancies are announced this week, the amount of the salary being placed in each case after the name of the institution :—

**Honorary Medical Officer.**

Westminster General Dispensary. Surgeon. Registered.

**Resident Medical Officers.**

Birmingham General Hospital. One Graduate.—£130.
Birmingham General Hospital. Two Assistant House Surgeons. Qualified.—No salary.
Leeds Borough Hospital. Superintendent. Doubly-qualified and registered.—£150.
London Fever Hospital, N. Assistant. Qualified.—£120.
Manchester, Pendlebury Hospital for Sick Children. Senior and Junior Resident Officers. Doubly-qualified.—£100 and £80.
Norwich Friendly Societies' Medical Institute. One.—220.
Ripon Dispensary. House Surgeon and Dispenser.—£100.

**Non-resident Medical Officer.**

Leeds, Yorkshire College. Demonstrator of Anatomy.—£150.

**Matrons.**

Dudley Guest Hospital. One.—£60.
Northwich Victoria Infirmary. One.

**Lady Probationers.**

Cork Hospital for Diseases of Women and Children. Two.

**Trained Nurses.**

Barton Regis Workhouse. Man as Head Nurse to Male Lunatics.—£30.
Bedford Infirmary. One.—£22.
Canterbury Hospital. Night Nurse.—£22.
Manchester Mauldeth Hospital for Incurables. Head.—£30.
Manchester Monsall Fever Hospital. Ward Sister.—£30.
Manchester Royal Eye Hospital. One.—£24.
Marylebone Western Dispensary. One.—£20.
West Bromwich District Hospital. Two. £22, rising to £25.

---

IT is proposed to erect a Cottage Hospital at Shooter's Hill, as a memorial of the Queen's Jubilee. By way of giving some immediate help to the movement, it has been resolved to hold a church parade of Friendly and Temperance Societies in St. Mary's Church, Woolwich, on the 24th inst., the proceeds of which will be devoted to the building fund.

---

ALTHOUGH Hospital Saturday is long since past in London, there are several country towns in which it is still looked forward to. Bolton, which has a good record for past years, has up to the present time contributed £1,047 2*s.* 4*d.* to the Saturday Fund. There are still many firms who have not yet sent in their books, and it may therefore be hoped that the total amount for the year will not only be equal to, but greater than that for any former year.

---

AT Wimborne Lady Glyn laid the fouudation-stone of a Cottage Hospital as a memorial of the year of Jubilee. The people of the district have entered with enthusiasm into the movement for building and endowing the institution, and there is little doubt that the hospital, commenced under such favourable auspices, will have a prosperous and useful history.

---

THE Hull Infirmary Working Men's Committee reports an increase of £56 in the amounts collected from the various workshops. We are glad to see working-men all over the country take up the hospital question with so much earnestness and success. Although hospitals are to a large extent supported by the wealthier classes, they are, after all, working-men's institutions, and should be so regarded by the working-men themselves.

---

A COTTAGE hospital is about to be built at Swindon, on a site generously given for that purpose. Collections are being made in all the villages round, and it is hoped that the hospital will be opened free of debt.

---

AT a meeting of the general committee of the Queen's Hospital, Birmingham, the Rev. J. C. Blissard, M.A., in the chair, a resolution was passed thanking the working-men of Birmingham for the liberal donation of £1,115 19*s.* 10*d.*, contributed by them for the funds of the hospital on Hospital Saturday, and also to the committee and officers of the movement for valuable aid.

---

THE Children's Hospital at Bradford has been removed to new temporary premises at Springfield Place, Manningham Lane. In consideration of the handsome donation of £400 by the widow and daughters of the late Mr. Benjamin Terry, the committee have named two cots respectively the "Benjamin Terry" Cot, and the "Mary Alice Terry" Cot.

---

AT Swansea the annual meeting of the General Hospital has just been held, under the presidency of Mr. Charles Bath. The hospital is exceedingly popular in the district, and as the report states, "for situation, airiness, and modern requirements is a model to the kingdom." The year began with a credit balance of £32 2*s.*, and ended with the still larger balance of £76 13*s.* The chairman, in moving the adoption of the report, expressed pleasure that the depression in trade had not seriously affected the funds of the hospital.

---

THE London School of Medicine for Women, 30, Henrietta Street, W.C., has been making wonderful progress of late, despite the want of encouragement which the general public have accorded to lady doctors. In India, where Mohamedan women have until recently been peculiar sufferers in consequence of their customs not allowing them to be seen by strange men, there is a wide sphere for useful work among lady doctors. Thirteen years ago a medical school for women was opened by Surgeon-General Balfour at Madras; later on, the Cama Hospital for Women was established at Bombay, and more recently still Lady Dufferin has carried out her beneficent scheme. The New Hospital for Women, in the Marylebone Road, where all the physicians are females, has now its out-patient department crowded daily—a proof that, at least among the poor, a feeling of confidence in the treatment of lady doctors has arisen. Mrs. Garrett Anderson, M.D., the Dean of the London School of Medicine for Women, stated at the annual prize distribution the other day, that while two years ago there were only thirty-three women studying medicine, there were now sixty six students attending the school. Of these, twenty-four were receiving clinical instruction at the Royal Free Hospital; twenty-three were preparing for the degrees of the University of London; five for the degrees of the Royal University of Ireland, and thirty-two for the licence of the Irish and Scotch Colleges.

At Sunderland the annual meeting in connection with the Hospital for Sick Children has just been held, under the presidency of Mr. Edwin Richardson, the Mayor. The report of the committee showed an expenditure of £409 3*s.* 9*d.*, and an income of £556 13*s.* 3*d.*, leaving a balance in favour of the institution of £147 11*s.* 6*d.*

---

The Devonshire Hospital, Buxton, has just issued its half-yearly report, giving evidence that the institution has been largely made use of during the last six months. The sum received on account of patients sent under the powers of the Governors of the Cotton Districts' Convalescent Fund has been considerably larger than that received during the corresponding half of last year. But the expenditure on the extension of the hospital beyond the really important sums granted by the Governors of the Cotton Districts' Fund still remains in a great measure a deduction from the investments of the hospital, thereby lessening its provision against emergencies in the future. This is very unfortunate for the hospital, and ought to have been prevented by the liberal help of the inhabitants of the district.

---

At South Shields, the secretaries of various Friendly Societies met under the presidency of the Mayor, Alderman Eltringham, for the purpose of arranging a demonstration and church parade in aid of the infirmary. One of the delegates asked what was the total expense of last year's demonstration, and remarked that the lodge with which he was connected were of opinion that not one-half of the amount collected went to the infirmary. Another delegate said that there was great feeling in the town about the excessive expenditure in connection with the last year's demonstration. A committee was formed to further the arrangements, and the meeting concluded with a vote of thanks to the Mayor for presiding.

---

In consequence of an outbreak of scarlet fever at Ingleston House School, Mr. Winstanley took away his four children after they had entered upon a new term, declining to pay the fees for that term. Miss Augusta Denman, the principal of the school, brought an action for the recovery of the fees, amounting to £41 6*s.* The judge decided that as the children had entered upon the term the parents were liable for the full amount claimed. This is an important case. Many parents are called upon in defence of the health of their children to take them away from school at a moment's notice, and on the first blush it would seem hard that on being obliged to take them away for no fault of their own, they should be compelled to pay. But what is merely hard to those who have to pay, would be ruinous to a school if it were decided that they could take their children away at a moment's notice and not pay for the remainder of the term. School establishments are kept open in the interest of the public, and the expenses of such establishments are continued whether there be an outbreak of fever or not. In spite therefore of an appearance of hardship, the judge's decision is undoubtedly in accordance with justice and common-sense.

Two babies' cots, with the requisite furnishings, have been given to the Johnson Hospital, Lincoln, by two gentlemen of the town. Those who have visited a children's hospital will be able to appreciate the value of such a gift.

---

The annual meeting of the friends and subscribers of the Woolton Convalescent Institution, Liverpool, was held there on Saturday afternoon. A number of ladies and gentlemen drove out from Liverpool, and were received and entertained by the Chairman and Treasurer, Mr. William Henry Tate, Miss Feek, the Lady Superintendent, and Mr. W. G. Johnson, Secretary. The institution, which is beautifully situated, and has large and well-kept gardens and grounds, is in a highly prosperous financial condition.

---

The Board of Management of the Grimsby Hospital were agreeably surprised on Friday to receive a deputation of four gentlemen, representing the "Brewers, Wine and Spirit Merchants, and Licensed Victuallers' Association," who presented to the hospital the handsome donation of £75. The deputation were shown over the wards and various departments of the hospital, and were much pleased with everything they saw.

---

Twenty thousand people gathered on Saturday in the Roundhay Park, Leeds, to hold high festival on behalf of the Working People's Hospital Fund movement. At Bradford the Whitsuntide galas have been found most valuable contributors to the funds of the medical charities, whilst at the same time providing a good deal of innocent amusement to the public. Swimming, boating, and a variety of athletic sports were arranged for at Roundhay, and were greatly enjoyed. We hope the results may be as financially satisfactory as the idea is undoubtedly good.

---

At the Royal Hospital for Incurables, Dublin, Her Majesty's Jubilee was celebrated by an entertainment provided for the inmates by the governors and other friends of the hospital. The band of the Black Watch attended from 2 till 4.30 p.m., and at intervals delighted the patients by the playing of a beautiful selection of music. A conjuring entertainment, a Punch-and-Judy show, and a variety of other sports were greatly enjoyed. Later in the afternoon tea and other refreshments were handed round, much to the pleasure and satisfaction of the patients. The proceedings terminated about 8 o'clock by the singing of "God Save the Queen."

---

At Northallerton, in Yorkshire, the annual sale of work and fancy articles in aid of the Cottage Hospital was recently held in the town-hall. The bazaar was opened by Mr. John Hutton, J.P., who said that the hospital had been established for eleven years, and had been a great blessing to the town. At present they were £160 in debt, and although the sum might be lessened by the end of the financial year, there would be a serious deficit. The landed proprietors, farmers, and others in this fox-hunting district of the North Riding should see to it that their most important local charity is not starved.

